# Relapse of Acute Myeloid Leukemia with t(16;21)(p11;q22) Mimicking Autoimmune Pancreatitis after Second Allogeneic Bone Marrow Transplantation

**DOI:** 10.5402/2011/285487

**Published:** 2011-01-10

**Authors:** Yuhei Kamada, Kazumi Suzukawa, Kenichi Taoka, Yasushi Okoshi, Yuichi Hasegawa, Shigeru Chiba

**Affiliations:** Department of Hematology, Graduate School of Comprehensive Human Sciences, University of Tsukuba, Tsukuba, Ibaraki 305-8575, Japan

## Abstract

We report the case of a 37-year-old woman who had a relapse of acute myeloid leukemia (AML) during treatment for chronic graft versus host disease (cGVHD) after allogeneic bone marrow transplantation. She was originally suspected of having autoimmune pancreatitis. Relapse of AML often occurs at extramedullary sites. Whereas the pancreas is rare as an organ of AML relapse, physicians should be aware that enlargement of the pancreas could be a sign of relapsed AML when excluding autoimmune pancreatitis, particularly during active cGVHD after allogeneic stem cell transplantation.

## 1. Introduction

Relapses of acute leukemia is devastating event after allogeneic hematopoietic stem cell transplantation. Some of them occur at extramedullary sites [1–3]. Diffuse infiltration of leukemia cells mimics inflammation of the organs. Chronic graft versus host disease (cGVHD) cause inflammation of organs and frequently resemble autoimmune diseases [4]. We report the case of extramedullary relapse of acute myeloid leukemia with t(16;21)(p11;q22) during treatment for cGVHD. This case was originally suspected of having autoimmune pancreatitis due to characteristic symptoms and radiologic findings of AIP.

## 2. Case Report

A 37-year-old woman was referred to Tsukuba University Hospital because of fever and pancytopenia in February 2003. The findings of a bone marrow aspiration and biopsy established a diagnosis of acute myeloid leukemia (AML) with maturation. Cytogenetic analysis of the leukemia cells revealed complex abnormalities involving t(16;21)(p11;q22) translocation. A FUS/TLS-ERG fusion transcript was detected by RT-PCR. The patient received cytarabine and daunorubicin induction chemotherapy and achieved complete remission after 2 courses of therapy. An allogeneic bone marrow transplantation (BMT) using bone marrow from an unrelated donor was performed at the first remission in October 2003. The conditioning regimen was Cyclophosphamide (60 mg/kg/day, day −6 and day −5) and total body irradiation (12 Gy/6 fr, day −4 to day −2). The FUS/TLS-ERG fusion transcript was undetectable after the BMT. Two years and eight months after the BMT, the patient experienced progressive appetite loss and underwent a gastrointestinal fiberscopic examination. A biopsy of the gastric mucosa revealed infiltration of leukemia cells. The bone marrow examination showed normal cellularity with increased blasts, and a diagnosis of relapsed AML was made in June 2006. She achieved a second remission after undergoing idarubicin and cytarabine reinduction therapy. A second unrelated BMT was performed in April 2007 from a donor different from the donor in the first BMT. The conditioning regimen was intravenous Busulfan (3.2 mg/kg/day, day −7 to −4), Cyclophosphamide (60 mg/kg/day, day −3), and antithymocyte globulin (15 mg/kg/day, day −2 and day −1).

**Figure 1:**
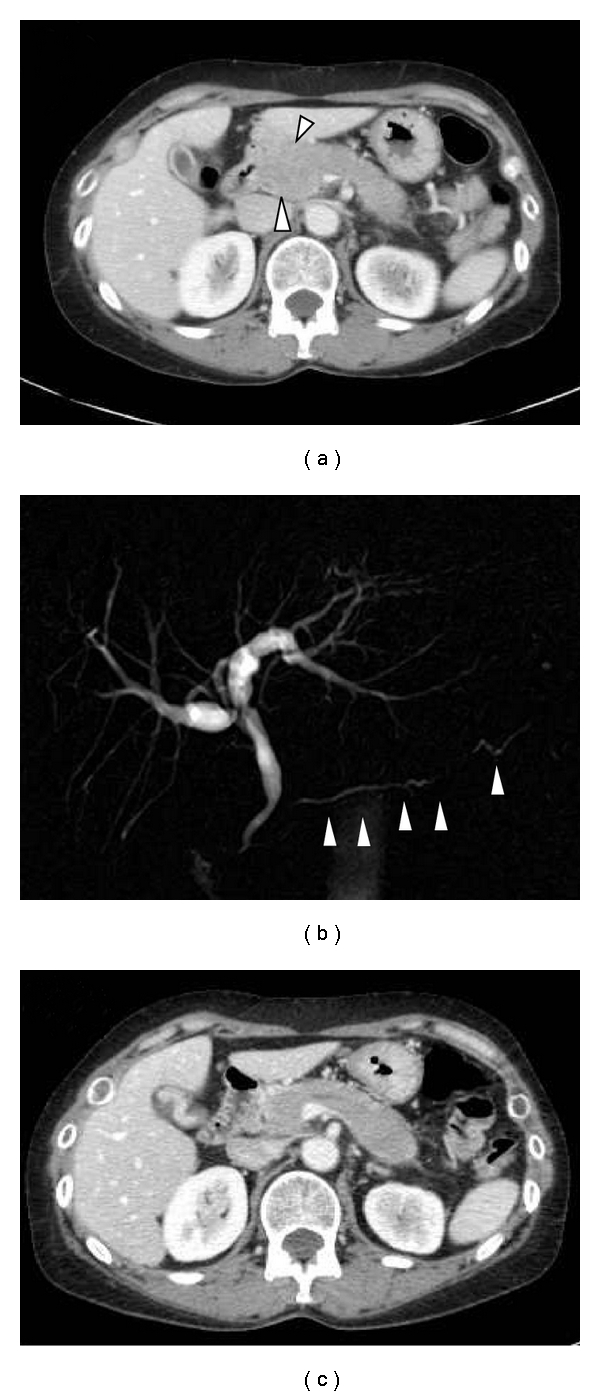
(a) CT scan of the upper abdomen. Arrowheads indicate swollen pancreas. (b) MRCP of the abdomen. Arrowheads indicate pancreatic duct. (c) CT scan after administration of prednisolone.

While the patient was suffering from cGVHD involving the skin and oral cavity, fever, epigastric pain, and diarrhea developed in July 2008. A mass was palpable in the upper abdomen. Serum amylase, aspartate aminotransferase (AST), alanine aminotransferase (ALT), and bilirubin levels were 327 IU/L, 1575 IU/L, 855 IU/L, and 1.6 mg/dL, respectively. No signs of relapse were noted in the bone marrow aspirate. A CT scan revealed diffuse swelling of the pancreas (Figure 1(a)). A magnetic resonance cholangiopancreatography (MRCP) revealed diffuse narrowing of the main pancreatic duct (Figure 1(b)), a finding consistent with autoimmune pancreatitis. A tentative diagnosis of pancreatitis associated with chronic GVHD was made. Administration of 1 mg/kg prednisolone and 1500 mg gabexate mesilate was started. Her symptoms improved and the serum amylase level returned to the normal range. The pancreas diminished in size, yet was still larger than normal (Figure 1(c)). Three months later, epigastralgia, jaundice, and elevated serum amylase flared up again. Blasts appeared in the peripheral blood and the bone marrow. A fiber gastroscopic study revealed an irregular thickening of the gastric mucosa, and a biopsy demonstrated an infiltration of leukemic cells. A CT scan detected multiple tumors in the anterior mediastinum, right atrium, stomach, and pancreatic head. Administration of gemtuzumab ozogamicin was temporarily effective for the epigastric pain. The patient chose palliative care and died of AML in June 2009.

## 3. Discussion

Pancreatitis has been reported in conjunction with acute GVHD [5, 6], but not with chronic GVHD. Chronic GVHD frequently resembles autoimmune diseases [4]. Autoimmune pancreatitis (AIP) is a well-recognized entity [7]. Characteristic radiologic findings of AIP made us administer steroids in the context of chronic GVHD. However, the serum level of IgG4, which is often elevated in autoimmune pancreatitis, was normal, and the pancreatitis recurred during the steroid maintenance therapy. Furthermore, a biopsy of the gastric mucosa, which was one of multiple tumors detected by the CT scan, demonstrated an infiltration of leukemic cells. We were not able to perform a histological study of the pancreas, but we eventually concluded that the pancreatitis was due to infiltration of leukemia cells rather than chronic GVHD- associated pancreatitis.

Around a quarter to a half of patients with acute leukemia relapse after allogeneic hematopoietic stem cell transplantation. Of those relapses, 15% to 23% occur at extramedullary sites without bone marrow relapse, although most patients eventually experience bone marrow relapse within a few months [1–3]. The relatively high frequency of isolated extramedullary relapse of AML is associated with the presence of chronic GVHD and the relapse at the later phases [3]. These facts are consistent with the speculation that the allogeneic immune attack is less effective in the extramedullary sites than in the bone marrow [1]. Sites of extramedullary relapse vary widely among patients, but the 2 most common sites are the central nervous system and the skin. Less common sites include the gastrointestinal tract, breast tissue, urogenital tract, paravertebral tissue, nasopharynx, paranasal sinus, peritoneal cavity, and pleura [8, 9]. To our knowledge, pancreatic swelling as a representation of AML relapse, particularly in the late phase after BMT, has not been reported.

The risk factors for extramedullary relapse based on leukemia cell characteristics have also been reported, including cytogenetic abnormalities such as t(8;21) and inv(16), expression of adhesion molecules such as CD56 (NCAM), expression of T cell markers such as CD2 and CD7, and FAB subtypes such as M2, M4, and M5 [10, 11]. In the present case, the leukemia cells were positive for CD56 but negative for CD2, CD4, and CD7. The cytogenetic study demonstrated t(16;21)(p11;q22) translocation as well as other complicated abnormalities. The t(16;21)(p11;q22) translocation results in the formation of the FUS/TLS-ERG fusion gene [12]. Acute myeloid leukemia with this translocation is known to be rare and associated with younger age at presentation. The median age at diagnosis is 22 years. Relapse is frequent in patients who have achieved complete remission, suggesting that this group of patients has a poor prognosis [13]. The FUS/TLS-ERG fusion gene consists of an RNA-binding protein, FUS, and a transcription factor, ERG. TLS-ERG expression alone induces a leukemogenic program in mice [14]. Previously, we reported the case of an AML patient with FUS/TLS-ERG who relapsed at the left femur after allogeneic BMT [15]. Interestingly, the same fusion gene is also detected in Ewing sarcoma [16]. Although an association between t(16;21)(p11;q22) translocation and extramedullary relapse has not been reported, it is interesting that the patients we reported here and previously had extramedullary relapse.

In summary, we have reported a case of a patient with relapsed AML with t(16;21)(p11;q22) who presented with pancreatitis as a representation of extramedullary relapse after a second allogeneic BMT. Extramedullary relapse of AML should be considered in the patient who presents pancreatitis-like signs and symptoms after BMT.
